# Peripheral immune cell imbalance is associated with cortical beta-amyloid deposition and longitudinal cognitive decline

**DOI:** 10.1038/s41598-023-34012-2

**Published:** 2023-05-31

**Authors:** Neel H. Mehta, Liangdong Zhou, Yi Li, Laura Beth McIntire, Anna Nordvig, Tracy Butler, Mony de Leon, Gloria C. Chiang

**Affiliations:** 1 Department of Biology, Cornell University, Ithaca, NY 14850 USA; 2 Brain Health Imaging Institute, Weill Cornell Medicine, NewYork-Presbyterian Hospital, 407 E 61st Street, New York, NY 10065 USA; 3 Department of Pathology and Cell Biology, Columbia University Medical Center, 630 West 168th Street, New York, NY 10065 USA; 4 Department of Neurology, Alzheimer’s Disease and Memory Disorders Program, Weill Cornell Medicine, NewYork-Presbyterian Hospital, 428 East 72nd Street Suite 500, New York, NY 10021 USA; 5 Department of Radiology, Division of Neuroradiology, Weill Cornell Medicine, NewYork-Presbyterian Hospital, 525 East 68th Street, Starr Pavilion, Box 141, New York, NY 10065 USA

**Keywords:** Dementia, Neurodegenerative diseases

## Abstract

Neuroinflammation is believed to be a key process in Alzheimer’s disease (AD) pathogenesis. Recently, the neutrophil-to-lymphocyte (NLR) and lymphocyte-to-monocyte ratios (LMR) have been proposed to be useful peripheral markers of inflammation. However, it is unclear how these inflammatory ratios relate to AD pathology, such as β-amyloid (Aβ) plaques and tau tangles. Using ^18^F-florbetapir and ^18^F-flortaucipir positron emission tomography (PET), we sought to determine how the NLR and LMR are associated with AD pathology both cross-sectionally and longitudinally. We further evaluated associations between the NLR and LMR and longitudinal cognitive decline. Using data from the Alzheimer’s Disease Neuroimaging Initiative, we analyzed blood, PET, and cognitive data from 1544 subjects—405 cognitively normal, 838 with mild cognitive impairment (MCI), and 301 with AD. Associations between the NLR and LMR and Aβ and tau on PET were assessed using ordinary least-squares and mixed-effects regression models, while adjusting for age, sex, years of education, and apolipoprotein E ε2 or ε4 carrier status. Associations between the NLR and LMR and cognitive function, as measured by the AD Assessment Scale-Cognitive Subscale, 13-item version, were also assessed. MCI and AD subjects had higher NLR (*p* = 0.017, *p* < 0.001, respectively) and lower LMR (*p* = 0.013, *p* = 0.023). The NLR, but not the LMR, was significantly associated with Aβ (*p* = 0.028), suggesting that higher NLR was associated with greater Aβ deposition in the brain. Neither the NLR nor the LMR was associated with tau deposition (*p* > 0.05). A higher NLR was associated with greater longitudinal cognitive decline (*p* < 0.001). A higher ratio of peripheral neutrophils to lymphocytes, possibly reflecting an imbalance in innate versus adaptive immunity, is related to greater Aβ deposition and longitudinal cognitive decline. As the field moves toward blood-based biomarkers of AD, the altered balance of innate versus adaptive immunity could be a useful biomarker of underlying pathology and may also serve as a potential therapeutic target.

## Introduction

Alzheimer’s disease (AD) is a widely prevalent, incurable neurodegenerative disease afflicting 5% of people between the ages of 65 and 74, 13% of people between the ages of 75 and 84, and 33% of people aged 85 and older^1^. Pathologically, it is characterized by beta-amyloid (Aβ) plaques and neurofibrillary tangles^2^. However, the underlying mechanisms that lead to these pathological lesions remain unclear.

Neuroinflammation has been implicated as an early process in AD pathogenesis^3^, with transgenic mouse models showing that elevated cytokines in the central nervous system lead to increased Aβ and tau deposition, triggering further cytokine production and a positive feedback loop that amplifies AD pathology^4,5^. Studies in human cohorts have also reported high levels of proinflammatory cytokines in the blood^6^ and cerebrospinal fluid (CSF)^7^ of patients with AD. Some studies have further reported that C-reactive protein and cytokines colocalize with neurofibrillary tangles^8^ and Aβ plaques^9^. Imaging of neuroinflammation has focused on microglial activation since microglia are considered the primary innate immune cells of the brain. One study using a tracer that targets the translocator protein of activated microglia (TSPO) on positron emission tomography (PET) showed that TSPO uptake localizes to areas of Aβ and tau deposition^10^.

Recently, quantitative ratios of peripheral immune cells have been shown to be useful biomarkers of systemic inflammation and altered immunity. Specifically, a higher neutrophil-to-lymphocyte ratio (NLR) has been associated with AD and all-cause dementia in many epidemiological studies^11–16^, although not in all^17^. However, whether the association between NLR and AD is mediated by Aβ and/or tau deposition is unclear. A decreased lymphocyte-to-monocyte ratio (LMR) has been associated with worse prognosis in patients with other neurological disorders, such as stroke^18^ and intracranial hemorrhage^19^. Neutrophils are typically considered markers of innate immunity, which are elevated with chronic inflammation, whereas lymphocytes are considered markers of adaptive immunity^20^. Therefore, alterations in the NLR and LMR may reflect an imbalance between innate and adaptive immunity. The use of ratios rather than the absolute number of neutrophils or lymphocytes could control for the effects of cross-subject variation.

Given the potential role of inflammation in AD, we sought to investigate the relationships among two peripheral immune cell ratios, NLR and LMR, and Aβ and tau deposition on PET, both cross-sectionally and longitudinally. We further explored whether alterations in these immune cell ratios are associated with longitudinal cognitive decline.

## Materials and methods

### Subject population

This study used data obtained from the Alzheimer’s Disease Neuroimaging Initiative (ADNI) online archive, which is publicly available at https://ida.loni.usc.edu/login.jsp^21^. The ADNI is a longitudinal, multicenter study of over 1500 adults across the United States and Canada, launched in 2003 to identify accurate imaging and fluid biomarkers of AD. The Principal Investigator of ADNI is Michael W. Weiner, MD, and is supported by the National Institute on Aging (NIA), the National Institute of Biomedical Imaging and Bioengineering (NIBIB), the Food and Drug Administration (FDA), private pharmaceutical companies, and non-profit organizations. All methods were carried out in accordance with relevant guidelines and regulations. Informed written consent was obtained from all participants at each site. Briefly, subjects were between the ages of 55 and 90, without clinical or structural evidence of a significant neurologic or psychiatric disease, and without systemic medical illness or laboratory abnormalities that would interfere with follow-up. Further details regarding inclusion and exclusion criteria can be found at www.adni-info.org.

One thousand five hundred and forty-four subjects—405 cognitively normal (NC), 838 with mild cognitive impairment (MCI), and 301 with AD—who had a baseline blood draw, after an overnight fast, were included in these analyses (Tables 1, 2). The NLR and LMR were calculated from the complete blood count. Cognitive function was assessed using the Alzheimer’s Disease Assessment Scale Cognitive Subscale (ADAS-Cog), 13-item version, which is the most widely used measure in clinical trials^22^. Of the 1544 subjects, 1434 had 1 follow-up ADAS-Cog assessment, 1110 had 2 assessments, 814 had 3 assessments, and 628 had 4 assessments, with a mean follow-up of 3.1 ± 2.9 years.

**Table 1:** Baseline characteristics of the overall cohort.

	Normal	Mild cognitive impairment (MCI)	Alzheimer’s disease (AD)	*p* value*
Number of subjects	405	838	301	
Age, years	75 [5.7]	73^†^ [7.6]	75 [7.6]	< 0.001
Sex, number of subjects (%)				
Male	204 (50%)	495 (59%)^†^	167 (55%)	0.015
Female	201 (50%)	343 (41%)	134 (45%)	
Education level, years	16.3 [2.7]	15.9^†^ [2.9]	15.1^†^ [3.0]	< 0.001
Number (%) of subjects with an APOE ε2 allele	55 (14%)	51 (6%)^†^	11 (4%)^†^	< 0.001
Number (%) of subjects with an APOE ε4 allele	109 (27%)	411 (49%)^†^	205 (68%)^†^	< 0.001
Neutrophils, ×10^9^/liter	3.92 [1.20]	4.05 [1.30]	4.36^†^ [1.39]	< 0.001
Lymphocytes, ×10^9^/liter	1.93 [2.04]	1.80 [0.68]	1.74^†^ [0.56]	0.09
Monocytes, ×10^9^/liter	0.40 [0.14]	0.41 [0.14]	0.41 [0.15]	0.57
Neutrophil-to-lymphocyte ratio	2.32 [1.00]	2.49^†^ [1.17]	2.74^†^ [1.30]	< 0.001
Lymphocyte-to-monocyte ratio	5.11 [3.35]	4.69^†^ [1.85]	4.57^†^ [1.67]	0.015
Baseline Alzheimer's Disease Assessment Scale Cognitive Subscale score	9.65 [5.12]	17.37^†^ [10.59]	31.37^†^ [8.46]	< 0.001

Data shown are means [standard deviations].

*p-values were obtained by the Kruskal–Wallis or Fisher’s Exact tests, depending on the variable type.

^†^Significantly different (*p* < 0.05) from the normal group, using the Wilcoxon Rank Sum Test or Fisher’s Exact test, depending on variable type.

**Table 2:** Baseline characteristics of the cohort that underwent florbetapir PET.

	Normal	Mild cognitive impairment (MCI), Amyloid PET negative	Mild cognitive impairment (MCI), Amyloid PET positive	Alzheimer’s disease (AD)	*p* value
Number of subjects	277	242	305	125	
Age, years	74 [5.8]	70^†^ [8.0]	73 [6.8]	75 [8.1]	< 0.001
Sex, number of subjects (%)					
Male	139 (50%)	135 (56%)	177 (58%)	75 (60%)	0.18
Female	138 (50%)	107 (44%)	128 (42%)	50 (40%)	
Education level, years	16.5 [2.6]	16.2 [2.5]	16.0^†^ [2.9]	15.7^†^ [2.6]	0.021
Number (%) of subjects with an APOE ε2 allele	34 (12%)	31 (13%)	6 (2%)^†^	5 (4%)^†^	< 0.001
Number (%) of subjects with an APOE ε4 allele	74 (27%)	53 (22%)	202 (66%)^†^	86 (69%)^†^	< 0.001
Neutrophils, ×10^9^/liter	3.9 [1.2]	3.9 [1.2]	4.1^†^ [1.3]	4.4^†^ [1.4]	< 0.001
Lymphocytes, ×10^9^/liter	1.8 [0.52]	1.9 [0.68]	1.7^†^ [0.60]	1.7 [0.58]	0.01
Monocytes, ×10^9^/liter	0.41 [0.14]	0.41 [0.14]	0.42 [0.15]	0.43 [0.16]	0.85
Neutrophil-to-lymphocyte ratio	2.3 [1.0]	2.6 [0.9]	2.7^†^ [1.3]	2.8^†^ [1.3]	< 0.001
Lymphocyte-to-monocyte ratio	4.9 [1.9]	4.9 [2.0]	4.5^†^ [1.8]	4.4^†^ [1.8]	0.01
Baseline Alzheimer's disease assessment scale total score	9.6 [5.1]	12.9^†^ [6.9]	18.8^†^ [9.5]	31.1^†^ [8.6]	< 0.001

Data shown are means [standard deviations]. *p*-values were obtained by the Kruskal–Wallis or Fisher’s Exact tests, depending on the variable type.

*p-values were obtained by the Kruskal–Wallis or Fisher’s Exact tests, depending on the variable type.

^†^Significantly different (*p* < 0.05) from the normal group, using the Wilcoxon Rank Sum Test or Fisher’s Exact test, depending on variable type.

### APOE genotyping

ADNI’s APOE genotyping was performed by the University of Pennsylvania Biofluid Bank Laboratory, using 6 ml of blood, within 24 h of collection, as previously described^23^.

### MR image acquisition

Structural three-dimensional T1-weighted MPRAGE sequences were obtained on all subjects on a 3 Tesla MRI, using a standardized, harmonized protocol (http://adni.loni.usc.edu/methods/documents/mri-protocols/).

### Amyloid and tau PET acquisition and analyses

^18^F-florbetapir SUVR values were processed by the ADNI PET imaging core and downloaded from the ADNI website (http://adni.loni.usc.edu/). ^18^F-florbetapir PET scans were coregistered to structural 3D T1 MPRAGE sequences and segmented as described previously^24^. ^18^F-florbetapir standardized uptake value ratios (SUVR) were obtained from a volume-weighted average of the mean uptake from cortical gray matter regions (lateral and medial frontal, anterior and posterior cingulate, lateral parietal, and lateral temporal) normalized to the cerebellum (white and gray matter). Centiloids were calculated as previously published^25^. The subgroup that underwent ^18^F-florbetapir PET scans consisted of 949 (61%) subjects: 277 NC, 547 MCI, and 125 AD. Six hundred forty-four had at least 1 follow-up ^18^F-florbetapir PET scan, 395 had 2 follow-up scans, 217 had 3 follow-up scans, 87 had 4 follow-up scans, and 5 had 6 follow-up scans, with a mean duration of follow-up of 3.1 ± 2.9 years. Two hundred twenty-five NC, 391 MCI, and 28 AD subjects had at least 1 follow-up ^18^F-florbetapir PET scan.

^18^F-flortaucipir PET SUVRs were processed by the ADNI PET imaging core and downloaded from the ADNI website. Briefly, scans were acquired as six frames of 5-min duration approximately 75 min after the injection of approximately 10 mCi of radiotracer. Frames were realigned and averaged. FreeSurfer (http://surfer.nmr.mgh.harvard.edu) segmentation^26^ was used to extract the mean SUVR in four regions-of-interest: (1) Braak 1 region (entorhinal cortex), (2) Braak 3 and 4 regions (parahippocampal gyri, fusiform gyri, lingual gyri, amygdala, middle and inferior temporal gyri, anterior/posterior/isthmus cingulate, insula, and temporal poles), (3) Braak 5 and 6 regions (superior/middle frontal gyri, lateral/medial orbitofrontal gyri, frontal poles, pars opercularis/orbitalis/triangularis, superior and inferior parietal lobules, lateral occipital lobes, supramarginal gyri, superior temporal gyri, precuneus, banks of the superior temporal sulcus, transverse temporal gyri), and (4) temporal regions (including the amygdala, entorhinal cortex, parahippocampal gyri, fusiform gyri, and inferior/middle temporal gyri)^27^. The inferior cerebellar cortex was used as the reference region in cross-sectional analyses, and the hemispheric white matter was used as the reference region in longitudinal analyses as recommended^28^. Braak 2 regions, including the hippocampi, were not included in the analyses due to potential contamination from off-target binding to the choroid plexus.

^18^F- flortaucipir PET scans were also processed by us using the standardized uptake value peak-alignment (SUVP) method^29^ to address potential variability in off-target binding in the cerebellar cortex reference region. The SUVP was computed voxel-by-voxel by subtracting the mode of the whole brain SUV, then dividing by the standard deviation of the whole brain SUV. Positive SUVP values in the medial temporal lobe were then averaged and included in the analyses. The subgroup that underwent ^18^F- flortaucipir PET scans consisted of 246 (16%) subjects: 117 NC, 127 MCI, and 2 AD subjects. One hundred sixteen had at least 1 follow-up ^18^F- flortaucipir PET scan, 47 had 2 follow-up scans, 12 had 3 follow-up scans, and 1 had 4 follow-up scans, with a mean duration of follow-up of 0.9 ± 1.1 years. Fifty-six NC, 60 MCI, and 0 AD subjects had at least 1 follow-up ^18^F- flortaucipir PET scan.

### Plasma cytokines

Five hundred thirty-eight subjects (35%) from our cohort underwent a plasma proteomics analysis using a 190-analyte multiplex immunoassay panel, developed on a Luminex xMAP platform by Rules-Based Medicine (RBM, Austin, TX)^30–32^. Briefly, 0.5 mL plasma samples were obtained after an overnight fast, then shipped frozen at − 80 degrees Celsius to RBM for analyses. The results were downloaded from the ADNI website in a file labeled “Biomarkers Consortium Plasma Proteomics Project RBM multiplex data,” and included measured values of IL-13, IL-16, IL-18, and TNFα. The levels of other plasma cytokines were below the threshold of detection, so results were not available for them.

### Statistical analysis

All statistical analyses were performed in STATA 16 (StataCorp, College Station, TX). Baseline differences across groups were assessed using the Kruskal–Wallis or Fisher’s Exact Tests, depending on the variable type. Differences between groups (i.e. NC vs. MCI, NC vs. AD) were than assessed using the Wilcoxon Rank Sum test.

To assess whether NLR and LMR were associated with the degree of cognitive impairment (NC, MCI, AD), we used multivariate regression analyses, including age, sex, APOE genotype, and education level as covariates. The NC was the reference group for the regression analyses. Post hoc, we included amyloid burden in centiloids to these regressions to see if the relationships between NLR/LMR and cognitive impairment were mediated by amyloid. We also included available IL-13, IL-16, IL-18, and TNFα levels in these regressions to investigate the potential role of peripheral cytokines.

We then assessed whether the NLR and LMR were associated with amyloid deposition on PET, again adjusting for age, sex, APOE genotype, education, and cognitive impairment. ^18^F-florbetapir SUVR was used as the outcome variable in the regression models.

To determine whether NLR and LMR were associated with longitudinal change in ^18^F-florbetapir SUVR, we used a linear mixed-effects model^33,34^ SUVR_ij_ = (B_0_ + β_0_) + β_1_ NLR_i_ + (β_2_ + β_3_ NLR_i_) t_ij_ + covariates + ε_ij_. SUVR_ij_ represents the SUVR of subject i at timepoint j, NLR_i_ represents the NLR of each subject, and t_ij_ represents the time interval between PET scans. (B_0_ + β_0_) are the coefficients for the random and fixed variations in baseline SUVR. The coefficient β_1_ represents the fixed effects of the association between NLR and SUVR at baseline. Finally, (β_2_ + β_3_) are the coefficients for time-dependent changes in SUVR, irrespective (β_2_) and respective (β_3_) of the NLR. The error term ε_ij_ represents random noise. The same covariates were included, as above.

Next, we used multivariate regression models to assess whether the NLR and LMR were associated with tau deposition on PET, in amyloid-positive individuals, defined as an SUVR > 1.11 or 20 centiloids^35^, using both the SUVR and SUVP in the aforementioned regions-of-interest as outcome variables. A linear mixed-effects model was used to assess whether the NLR and LMR were associated with longitudinal change in ^18^F- flortaucipir SUVR and SUVP: SUVR_ij_ or SUVP_ij_ = (B_0_ + β_0_) + β_1_ NLR_i_ + (β_2_ + β_3_ NLR_i_) t_ij_ + covariates + ε_ij_.

Finally, we used multivariate regression models to assess whether the NLR and LMR were associated with sum of scores on the ADAS-Cog. A linear mixed-effects model was used to assess whether the NLR and LMR were associated with longitudinal change in ADAS-Cog: ADAS-Cog Score_ij_ = (B_0_ + β_0_) + β_1_ NLR_i_ + (β_2_ + β_3_ NLR_i_) t_ij_ + covariates + ε_ij_. For all regression models, plots of residuals were assessed for normality, and quantiles of variable transforms were assessed to better fit a normal distribution. A p-value of less than 0.05 was considered significant.

### Exclusion of systemic inflammatory disorders and medications

In post hoc analyses, we downloaded the medical histories (RECMHIST.csv file) and medication lists (RECCMEDS.csv file) from the ADNI online database for the subjects in these analyses. We then carefully reviewed these lists and identified 129 subjects with systemic inflammatory disorders that could peripheral blood counts, including inflammatory bowel disease, rheumatoid arthritis, myocarditis, pericarditis, asthma, and lupus. Next, we identified 109 subjects who were on medications that could affect peripheral blood counts (2 on lithium, 107 on steroids).

### Ethical approval

The ADNI database used in these analyses is de-identified and publicly available. As a result, the Weill Cornell Medicine Institutional Review Board determined that the use of this database for this study does not constitute human subjects research, and IRB approval was not required.

## Results

Baseline characteristics and group comparisons for the overall cohort are shown in Table 1. Compared to the NC group, the MCI group was younger (mean ± SD: MCI 73 ± 7.6 vs. NC 75 ± 5.7 years, *p* < 0.001) and had a higher proportion of males (MCI 59% male vs NC 50% male, *p* = 0.004). The MCI and AD groups had fewer years of education (MCI 15.9 ± 2.9 vs. NC 16.3 ± 2.7 years, *p* = 0.03; AD 15.1 ± 3.0 vs NC, *p* = 0.003), fewer APOE ε2 carriers (MCI 6% vs. NC 14%, *p* < 0.001; AD 4% vs. NC, *p* < 0.001), and more APOE ε4 carriers (MCI 49% vs. NC 27%, *p* < 0.001; AD 68% vs. NC, *p* < 0.001) than the NC group. The MCI and AD groups also had higher NLR (MCI 2.49 ± 1.17 vs. NC 2.32 ± 1.00, *p* = 0.02; AD 2.74 ± 1.30 vs. NC, *p* < 0.001), lower LMR (MCI 4.69 ± 1.85 vs. NC 5.11 ± 3.35, *p* = 0.008; AD 4.57 ± 1.67 vs. NC, *p* < 0.001), and higher ADAS-Cog scores (MCI 17.37 ± 10.59 vs. NC 9.65 ± 5.12, *p* < 0.001; AD 31.37 ± 8.46 vs. NC, *p* < 0.001) than the NC group.

Baseline characteristics and group comparisons for the subgroup that underwent amyloid PET are shown in Table 2. Compared to the NC group, the amyloid-negative MCI group was younger (MCI 70 ± 8.0 vs. NC 74 ± 5.8 years, *p* < 0.001) and had higher ADAS-Cog scores (MCI 12.9 ± 6.9 vs. NC 9.6 ± 5.1, *p* < 0.001). The amyloid-positive MCI group had fewer years of education (MCI 16.0 ± 2.9 vs. NC 16.5 ± 2.6 years, *p* = 0.03), fewer APOE ε2 carriers (MCI 2% vs. NC 12%, *p* < 0.001), more APOE ε4 carriers (MCI 66% vs. NC 27%, *p* < 0.001), higher NLR (MCI 2.7 ± 1.3 vs. NC 2.3 ± 1.0, *p* = 0.002), lower LMR (MCI 4.5 ± 1.8 vs. NC 4.9 ± 1.9, *p* = 0.01), and higher ADAS-Cog scores (MCI 18.8 ± 9.5 vs. NC 9.6 ± 5.1, *p* < 0.001).

### MCI and AD were independently associated with higher NLR and lower LMR

Having MCI (coefficient ± SE 0.16 ± 0.071, *p* = 0.021) or AD (coefficient 0.35 ± 0.091, *p* < 0.001) was independently associated with a higher NLR (Table 3). The results remained significant using a log transform of NLR (*p* = 0.017, *p* < 0.001, respectively). Older age, male sex, and carrying an APOE ε4 allele were also associated with a higher NLR in multivariate regressions (*p* < 0.05). In the subgroup that underwent amyloid PET, the MCI group with a positive amyloid PET scan had a higher NLR than the NC group (coefficient 0.33 ± 0.10, *p* = 0.001), but the amyloid-negative MCI group did not (coefficient 0.26 ± 0.10, *p* = 0.80). The results did not change using a log transform of NLR (*p* = 0.002, *p* = 0.66, respectively).

**Table 3:** Multivariate regression models demonstrating baseline predictors of the neutrophil-to-lymphocyte and lymphocyte-to-monocyte ratios.

	Regression coefficient [95% CI]	*p*-value
Neutrophil-to-lymphocyte ratio		
Age	0.023 [0.015, 0.031]	< 0.001*
Male	0.15 [0.036, 0.27]	0.01*
Years of education	0.020 [− 0.00085, 0.040]	0.06
Mild cognitive impairment	0.16 [0.024, 0.30]	0.021*
Alzheimer’s disease	0.35 [0.18, 0.53]	< 0.001*
APOE ε2 allele	− 0.097 [− 0.32, 0.13]	0.40
APOE ε4 allele	0.19 [0.066, 0.31]	0.003*
Lymphocyte-to-monocyte ratio		
Age	− 0.033 [− 0.049, − 0.017]	< 0.001*
Male	− 0.75 [− 0.98, − 0.52]	< 0.001*
Years of education	− 0.038 [− 0.078, 0.0029]	0.069
Mild cognitive impairment	− 0.36 [− 0.63, − 0.081]	0.011*
Alzheimer’s disease	− 0.43 [− 0.78, − 0.074]	0.018*
APOE ε2 allele	0.63 [0.18, 1.07]	0.006*
APOE ε4 allele	− 0.12 [− 0.36, 0. 13]	0.34

*p < 0.05.

In post hoc mediation analysis in the subgroup that underwent amyloid PET, the associations between NLR and MCI (coefficient ± SE 0.19 ± 0.087, *p* = 0.032) and AD (coefficient 0.38 ± 0.13, *p* = 0.003) were attenuated after inclusion of amyloid burden in centiloids (MCI coefficient 0.16 ± 0.088, *p* = 0.073; AD coefficient 0.30 ± 0.13, *p* = 0.027), but remained significant in the AD group. This suggests that the association between the NLR and MCI is mediated by amyloid, and the association between the NLR and AD is partially mediated by amyloid.

Having MCI (coefficient − 0.36 ± 0.14, *p* = 0.011) or AD (coefficient -0.43 ± 0.18, *p* = 0.018) was independently associated with a lower LMR (Table 3). The results remained significant using a log transform of LMR (*p* = 0.013, *p* = 0.023, respectively). Older age, male sex, and carrying an APOE ε2 allele were also associated with the LMR (*p* < 0.05). In the subgroup that underwent amyloid PET, the MCI group with a positive amyloid PET scan had a lower LMR than the NC group (coefficient − 0.37 ± 0.16, *p* = 0.02), but the amyloid-negative MCI group did not (coefficient − 0.081 ± 0.16, *p* = 0.62). The results did not change using a log transform of LMR (*p* = 0.008, *p* = 0.45, respectively).

IL-13, IL-16, IL-18, and TNFα cytokine levels were not significantly associated with the NLR or LMR (*p* > 0.05).

### The NLR but not the LMR was associated with baseline amyloid deposition on PET

**Figure 1:**
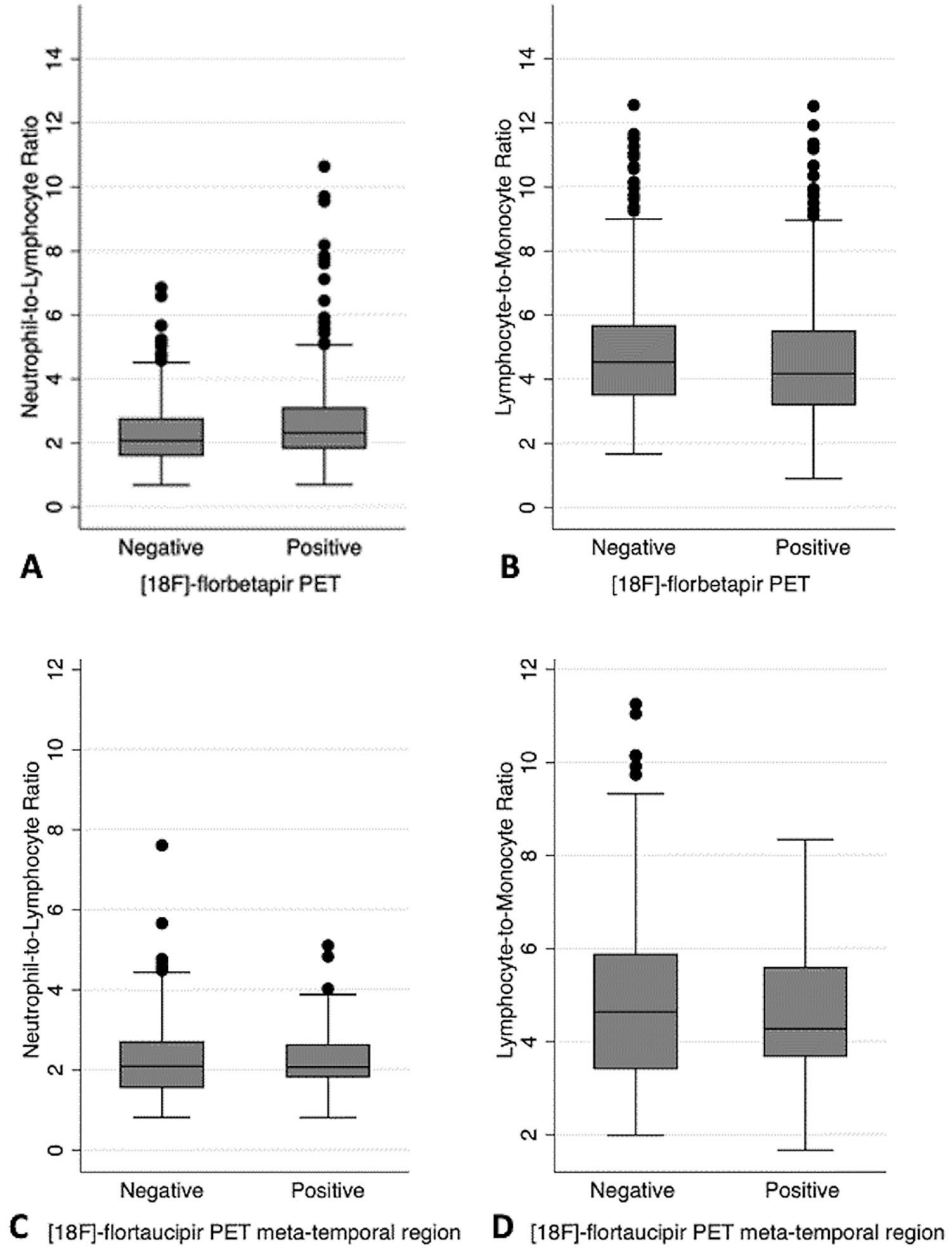
Boxplots showing the relationships among ^18^F-florbetapir PET, ^18^F-flortaucipir PET, the neutrophil-to-lymphocyte ratio (NLR), and the lymphocyte-to-monocyte (LMR) ratio. Subjects with a positive ^18^F-florbetapir PET scan had higher NLR (*p* < 0.001) (**A**) and lower LMR (*p* = 0.002) (**B**). A positive ^18^F-flortaucipir PET scan in the meta-temporal region was not associated with a higher nor lower NLR (**C**) or LMR (**D**) (*p* > 0.05).

**Figure 2:**
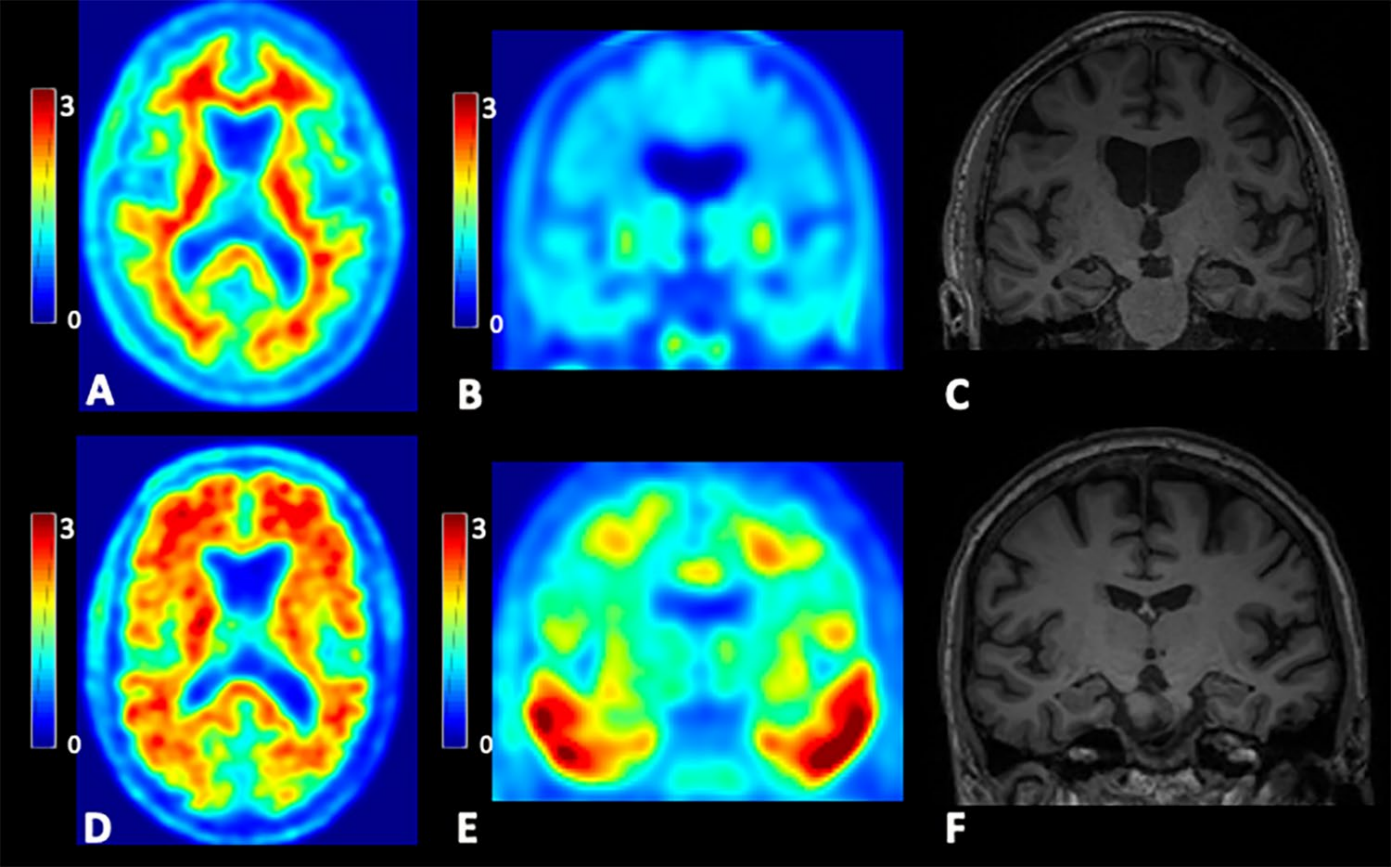
Axial ^18^F-florbetapir PET, coronal ^18^F-flortaucipir PET and coronal T1-weighted MR images from two subjects with mild cognitive impairment. SUVR images were intensity normalized by the cerebellar cortex. Subject 1 is a 75 year-old man with a global SUVR of 1.03 on ^18^F-florbetapir PET (**A**), consistent with a negative amyloid scan, a negative ^18^F-flortaucipir PET scan (**B**), and mild ventricular enlargement on MRI (**C**), suggestive of mild volume loss. His neutrophil-to-lymphocyte ratio (NLR) was 1.04, and his lymphocyte-to-monocyte ratio was 6.5. Subject 2 is a 74 year-old man with a global SUVR of 1.60 on ^18^F-florbetapir PET (**D**), consistent with a positive amyloid scan, a positive ^18^F-flortaucipir PET scan (**E**), and no significant volume loss on MRI (**F**). His NLR was 3.8, higher than Subject 1, and his LMR was 2.29, lower than Subject 1.

**Figure 3:**
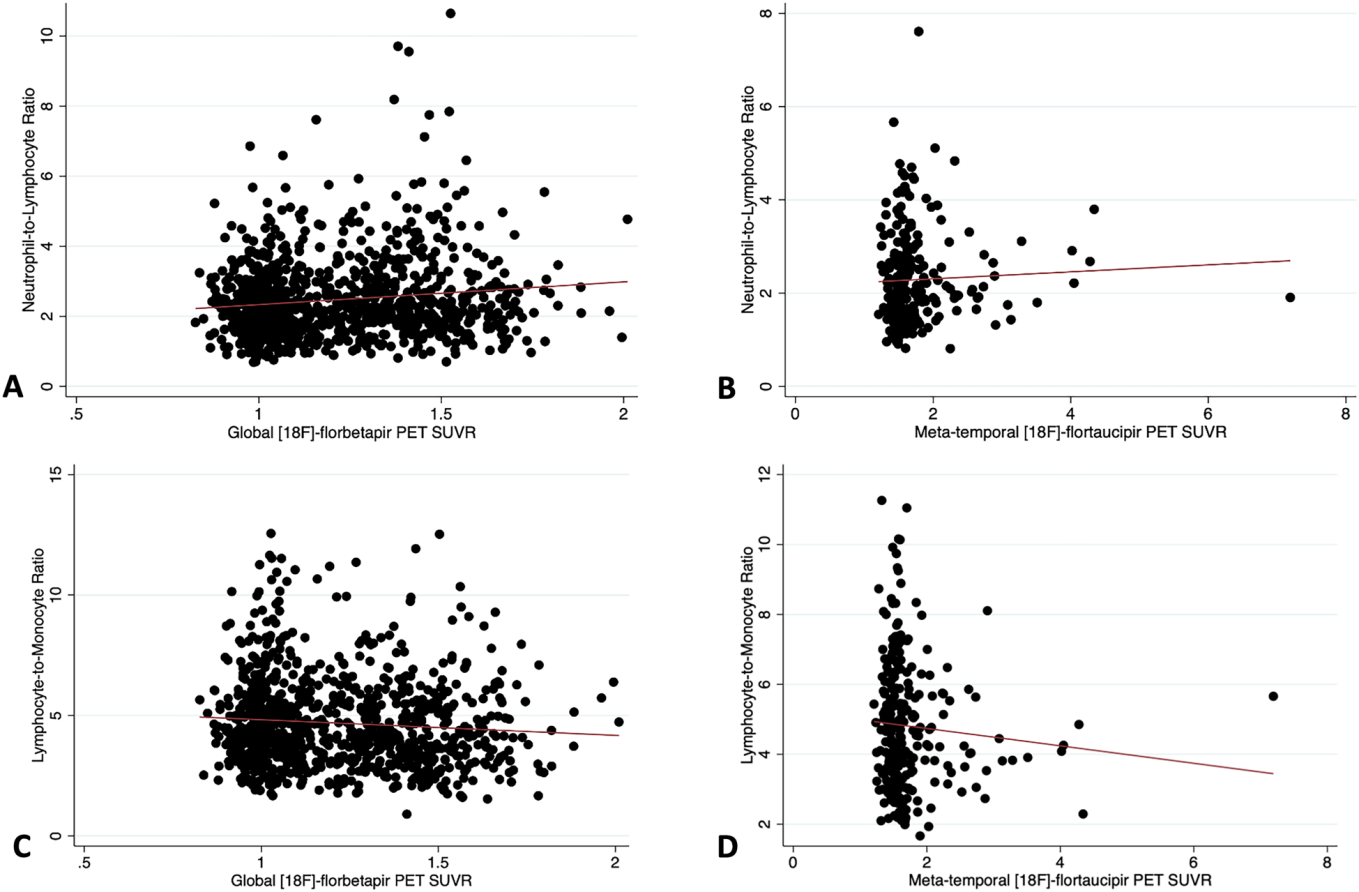
Scatterplots demonstrating the relationships among global ^18^F-florbetapir PET SUVR, ^18^F-flortaucipir PET SUVR in the meta-temporal regions, the neutrophil-to-lymphocyte ratio (NLR), and the lymphocyte-to-monocyte (LMR) ratio. Global ^18^F-florbetapir PET SUVR was significantly associated with the NLR (*p* = 0.047) (**A**), but not the LMR (*p* = 0.15) (**C**). ^18^F-flortaucipir PET SUVR in the meta-temporal regions was not significantly associated with the NLR (*p* = 0.5) (**B**) or LMR (*p* = 0.8) (**D**).

A positive amyloid PET scan was associated with a higher NLR (coefficient 0.35 ± 0.075, *p* < 0.001) (Figs. 1 and 2). This positive association between the global amyloid levels in centiloids and NLR (coefficient 0.0026 ± 0.00094, *p* = 0.005) persisted after adjusting for age, sex, years of education, and APOE 2 or 4 carrier status (Fig. 3, Table 4). The results remained significant using a log transform of NLR (p = 0.015). The NLR was not associated with longitudinal change in amyloid burden in centiloids (coefficient 0.030 ± 0.090, *p* = 0.74).

**Table 4:** Regression coefficients demonstrating the associations between amyloid deposition on [18F]-florbetapir PET and neutrophil-to-lymphocyte and lymphocyte-to-monocyte ratios.

	Regression coefficient [95% CI]	*p*-value
Neutrophil-to-lymphocyte ratio		
Amyloid PET (centiloids)	0.0026 [0.00078, 0.0045]	0.005*
Age, years	0.022 [0.011, 0.032]	< 0.001*
Male	0.22 [0.064, 0.37]	0.005*
Years of education	0.027 [− 0.00099, 0.055]	0.06
APOE ε2 allele	− 0.0039 [− 0.28, 0.27]	0.98
APOE ε4 allele	0.12 [− 0.044, 0.29]	0.15
Lymphocyte-to-monocyte ratio		
Amyloid PET in centiloids	− 0.0027 [− 0.0056, 0.00016]	0.065
Age, years	− 0.038 [− 0.055, − 0.022]	< 0.001*
Male	− 0.92 [− 1.16, − 0.68]	< 0.001*
Years of education	− 0.055 [− 0.098, − 0.012]	0.013*
APOE ε2 allele	0.26 [− 0.17, 0.69]	0.24
APOE ε4 allele	− 0.039 [− 0.30, 0.22]	0.77

A positive amyloid PET scan was associated with a lower LMR (coefficient =  − 0.37 ± 0.12, *p* = 0.002) (Figs. 1 and 2). However, this negative association between the global amyloid levels in centiloids and LMR was no longer significant (coefficient − 0.0027 ± 0.0015, *p* = 0.065) after adjusting for covariates (Fig. 3). When using the log transform of the LMR for normality, the association between amyloid levels in centiloids and LMR became significant (*p* = 0.024), even after including covariates. The LMR was not associated with longitudinal change in amyloid levels in centiloids (coefficient − 0.018 ± 0.044, *p* = 0.68).

### Neither the NLR nor the LMR was associated with regional tau deposition PET

Cross-sectionally, the NLR was not associated with tau SUVR in the four a priori defined regions-of-interest (metatemporal coefficient − 0.060 ± 0.088, *p* = 0.50; Braak 1 region coefficient − 0.042 ± 0.094, *p* = 0.66; Braak 3 and 4 regions coefficient − 0.040 ± 0.063, *p* = 0.53; Braak 5 and 6 regions coefficient − 0.022 ± 0.039, *p* = 0.57) (Figs. 1 and 3, Table 5). After using the inverse square for improving normality of the tail of the distribution, the associations remained nonsignificant (metatemporal *p* = 0.30, Braak 1 *p* = 0.99, Braak 3 and 4 *p* = 0.41, Braak 5 and 6 *p* = 0.50).

**Table 5:** Regression coefficients demonstrating the associations between tau deposition on [18F]-flortaucipir PET and neutrophil-to-lymphocyte and lymphocyte-to-monocyte ratios.

	Regression coefficient [95% CI]	*p*-value
Neutrophil-to-Lymphocyte Ratio		
Regional tau PET SUVR in meta-temporal regions	− 0.060 [− 0.23, 0.11]	0.50
Regional tau PET SUVR in Braak 1 regions	− 0.042 [− 0.23, 0.15]	0.66
Regional tau PET SUVR in Braak 3 and 4 regions	− 0.040 [− 0.16, 0.085]	0.53
Regional tau PET SUVR in Braak 5 and 6 regions	− 0.022 [− 0.10, 0.055]	0.57
Regional tau PET SUVP in the medial temporal regions	− 0.015 [− 0.100, 0.071]	0.73
Lymphocyte-to-Monocyte ratio		
Regional tau PET SUVR in meta-temporal regions	− 0.018 [− 0.15, 0.12]	0.80
Regional tau PET SUVR in Braak 1 regions	− 0.049 [− 0.20, 0.097]	0.51
Regional tau PET SUVR in Braak 3 and 4 regions	− 0.014 [− 0.11, 0.083]	0.77
Regional tau PET SUVR in Braak 5 and 6 regions	− 0.027 [− 0.087, 0.034]	0.38
Regional tau PET SUVP in the medial temporal regions	− 0.0030 [− 0.065, 0.059]	0.92

*SUVR* standardized uptake value ratio.

*SUVP* standardized uptake value peak alignment.

Values shown are regression coefficients [95% CI] (*p*-values). Regression models included age, sex, years of education, cognitive status, and APOE genotype as covariates.

Longitudinally, the NLR was not associated with change in tau SUVR (metatemporal coefficient 0.0035 ± 0.013, *p* = 0.79; Braak 1 region coefficient -0.0045 ± 0.021, *p* = 0.83; Braak 3 and 4 regions coefficient 0.0027 ± 0.011, *p* = 0.81; Braak 5 and 6 regions coefficient 0.0060 ± 0.012, *p* = 0.61). After using the inverse square for improving normality of the tail of the distribution, the associations remained nonsignificant (metatemporal *p* = 0.94, Braak 1 *p* = 0.23, Braak 3 and 4 *p* = 0.99, Braak 5 and 6 *p* = 0.50).

Cross-sectionally, the LMR was likewise not associated with tau SUVR cross-sectionally (metatemporal coefficient − 0.018 ± 0.069, p = 0.80; Braak 1 coefficient − 0.049 ± 0.073, *p* = 0.51; Braak 3 and 4 regions coefficient − 0.014 ± 0.049, *p* = 0.77; Braak 5 and 6 regions coefficient − 0.027 ± 0.030, *p* = 0.38) (Table 5). After using the inverse square for improving normality of the tail of the distribution, the associations remained nonsignificant (metatemporal *p* = 0.97, Braak 1 *p* = 0.35, Braak 3 and 4 *p* = 0.85, Braak 5 and 6 regions *p* = 0.59).

Longitudinally, the LMR was not associated with change in tau SUVR (metatemporal coefficient 0.0057 ± 0.0075, *p* = 0.44; Braak 1 region coefficient 0.00057 ± 0.012, *p* = 0.96; Braak 3 and 4 regions coefficient 0.0040 ± 0.0064, *p* = 0.53; Braak 5 and 6 regions coefficient 0.0020 ± 0.0068, *p* = 0.76). After using the inverse square for improving normality of the tail of the distribution, the associations remained nonsignificant (metatemporal *p* = 0.50, Braak 1 *p* = 0.35, Braak 3 and 4 *p* = 0.47, Braak 5 and 6 *p* = 0.99).

The NLR was also not associated with tau SUVP on PET in the medial temporal lobe, cross-sectionally (coefficient − 0.0099 ± 0.039, *p* = 0.80) or longitudinally (coefficient − 0.0020 ± 0.0055, *p* = 0.71). The LMR was also not associated with tau SUVP on PET in the medial temporal lobe, cross-sectionally (coefficient − 0.0030 ± 0.031, *p* = 0.92) or longitudinally (coefficient -0.000038 ± 0.0025, *p* = 0.99).

### The NLR was associated with longitudinal change in the ADAS-Cog score

At baseline, neither the NLR (coefficient = 0.37 ± 0.24, *p* = 0.13) nor the LMR was associated with the ADAS-Cog score (coefficient =  − 0.27 ± 0.15, *p* = 0.075). Using a square-root transformation for normality, the NLR remained not significantly associated with the ADAS-Cog score (*p* = 0.13), but the LMR because associated with the ADAS-Cog (*p* = 0.03), such that a lower LMR was associated with a higher ADAS-Cog score.

The NLR was associated with longitudinal change in the ADAS-Cog score (coefficient 0.22 ± 0.048, *p* < 0.001). Each unit increase in the NLR was associated with a 0.50 point increase per year in the ADAS score. This remains significant using the square-root transformation for normality (*p* = 0.001). There was no significant association between the LMR and longitudinal change in ADAS (coefficient − 0.014 ± 0.028, *p* = 0.62), and this stayed nonsignificant using the square-root transformation for normality (*p* = 0.62).

### Excluding subjects with systemic inflammatory disorders and medications did not significantly change the results

After excluding the 129 subjects with systemic inflammatory disorders that could peripheral blood counts, the results did not significantly change. The NLR remained associated with MCI (coefficient 0.18 ± 0.092, *p* = 0.048), amyloid-positive MCI (coefficient 0.32 ± 0.11, *p* = 0.002), and AD (coefficient 0.36 ± 0.13, *p* = 0.008). The LMR remained associated with MCI (coefficient − 0.32 ± 0.15, *p* = 0.036), amyloid-positive MCI (coefficient − 0.34 ± 0.17, *p* = 0.04), and AD marginally (coefficient − 0.37 ± 0.19, *p* = 0.05). The NLR remained significantly associated with the burden of amyloid in centiloids (coefficient 0.0026 ± 0.0010, *p* = 0.009), and the LMR remained not significantly associated with baseline amyloid (coefficient -0.0025 ± 0.0016, *p* = 0.11). Both the NLR and LMR remained not significantly associated with longitudinal change in amyloid, baseline tau deposition, longitudinal change in tau, or baseline ADAS-Cog (*p* > 0.05). The NLR, but not the LMR, remained significantly associated with longitudinal change in ADAS-Cog (coefficient 0.47 ± 0.09, *p* < 0.001).

After excluding the 109 subjects on steroids or lithium, which could affect peripheral blood counts, the LMR was found to be associated with baseline ADAS-Cog score (coefficient − 0.31 ± 0.16, *p* = 0.047), such that a lower LMR was associated with a higher ADAS-Cog score, or more clinical impairment. All other associations did not change.

## Discussion

The major findings of our study were: (1) MCI and AD were both associated with a higher NLR and lower LMR, (2) the NLR, but not the LMR, was significantly associated with higher baseline Aβ on PET and longitudinal change in ADAS, and (3) neither NLR or LMR was associated with tau deposition on PET cross-sectionally or longitudinally. Taken together, our analysis suggests that alterations in the balance of peripheral neutrophils and lymphocytes, as described by the NLR, possibly reflecting altered innate versus adaptive immunity, are related to Aβ deposition and longitudinal cognitive change.

Our first major finding was that MCI and AD groups had higher NLR and lower LMR values. The finding of higher NLR in MCI and AD is concordant with prior studies that reported higher NLR in AD^12–16^ and all-cause dementia^11^, although it is discordant with one prior study that did not find elevated NLR in AD^17^. Notably, we found that this higher NLR was independent of other baseline variables, such as age^36^, male sex^14^, and APOE4 carrier status^14^. Our finding of higher NLR and lower LMR can be explained by a higher neutrophil and/or lower lymphocyte count in the setting of MCI and AD, both of which were observed in our cohort (Tables 1, 2).

A higher neutrophil count in MCI and AD can be a marker of an activated innate immune system in the periphery. In AD, prolonged activation of microglia in the central nervous system has been associated with higher levels of proinflammatory cytokines in the periphery, including interleukin-1, interleukin-1B, and tumor necrosis factor-alpha (TNF-α), implying immune cell activation beyond the blood–brain barrier (BBB)^37^. TNF-α can further induce proliferation of neutrophils via a survival effect that is mediated via release of interleukin-9 through an NF-kB dependent pathway^38^. Dying neurons in the central nervous system may also relay signals to stimulate peripheral inflammation^39,40^. Together, the activated microglia, increased cytokine levels, and increased neutrophil count reflect an activated innate immune response. Furthermore, in a population-based study, individuals with dementia have been shown to have trouble with immune resolution, resulting in an inability to halt the acute phase of inflammation and clear these recruited neutrophils^12^.

A decreased lymphocyte count in MCI and AD may be explained by several mechanisms. First, activated neutrophils can release enzymes and inflammatory mediators that suppress lymphocyte activation in the blood. For example, neutrophils can release proteases, that cleave interleukin-2 and interleukin-6 receptors from the surface of T lymphocytes^41^, and the enzyme, arginase 1, which depletes the environment of arginine and downregulates T cells^42^. Activated neutrophils can also suppress T lymphocyte activity via release of reactive oxygen species (ROS) and altered cell adhesion processes^43,44^. Activated neutrophils can also redirect lymphocytes from the periphery to the CNS, via upregulation of matrix-metalloproteinase 9, disrupting the blood–brain barrier^45,46^ and allowing lymphocytes to migrate into the CNS. Activation of lymphocytes in the CNS may also occur via upregulation of the genes, CD83 and TAP1^47^. These immunological mechanisms may explain the higher NLR and lower LMR values in MCI and AD.

Our second major finding that the NLR was significantly associated with Aβ, but not tau, deposition on PET suggests that these alterations in neutrophils and lymphocytes occur in concert with amyloid, early in AD pathogenesis, prior to accumulation of tau pathology. One prior study showed a weak association between NLR and Aβ deposition on PET cross-sectionally, although not longitudinally, which is concordant with our study^14^. Another study showed an association between the NLR and decreased CSF levels of Aβ, which typically reflect increased Aβ binding in the brain^48^. Pathologically, many studies have found activated microglia in close proximity to Aβ plaques^49^, possibly due to the role of these microglia in phagocytosing amyloid plaques to clear them from the central nervous system^50^. Two prior studies using TSPO tracers to assess microglial activation in the brain found associations with Aβ deposition on PET^9^, although a third did not^51^. As described previously, these activated microglia then increase proinflammatory cytokines in the periphery. In turn, proinflammatory cytokines can further increase Aβ deposition. For example, interleukin-1 has been shown to activate a protein kinase C-mediated pathway, activating expression of APP and increasing Aβ levels^52^, and inhibition of IL-1beta in the periphery has been shown to reduce Aβ levels^53^. Similarly, interleukin-1b and TNF-α can enhance the activity of gamma-secretase, increasing the cleavage amyloid precursor protein to Aβ^54^. This feedback loop of Aβ deposition, activated microglia, and increased cytokines further induce neutrophil proliferation^37^, resulting in a higher NLR. The neutrophils that localize near Aβ plaques also promote the release of neutrophil extracellular traps, via an LFA-1 integrin mediated pathway, contributing to neuronal death^55^, further stimulating peripheral inflammation^36,37^. Other studies have shown that as Aβ deposition increases in the precuneus of the brain, lymphocytes shift from naïve to memory B cells, leading to decreased lymphocytes in the periphery and increased lymphocytes in the central nervous system^56,57^ adding to an elevated peripheral NLR.

Notably, the NLR was associated with greater cognitive decline longitudinally, but not increased longitudinal Aβ or tau deposition. This suggests that the effects of Aβ plaques, microglial activation, and systemic inflammation occur early in disease progression. Later in the disease course, systemic inflammation may play a role in non-Aβ, non-tau disease mechanisms.

Several limitations of our study should be considered. The main limitation was the retrospective nature of the study, using data from the ADNI rather than a community-based cohort with potential comorbidities, which could affect peripheral inflammatory markers in various ways. Secondly, less than half of the subjects had undergone tau PET scans, with a smaller fraction having undergone follow-up tau PET scans, resulting in a smaller sample size for those analyses. Finally, microglial function was not directly assessed in this study, since TSPO tracer PET scans were not performed in the ADNI. Nevertheless, our study provides strong evidence of alterations in peripheral immune cell ratios in MCI and AD, related to AD pathology and cognitive decline.

## Conclusions

As the field moves toward blood-based markers of AD, systemic markers of inflammation are highly promising biomarkers and potential therapeutic targets for disease modification. Our study provides strong evidence for a higher NLR and lower LMR in MCI and AD, partially mediated by Aβ pathology. Future community-based, prospective studies may assess the direct role of microglial activation and cytokine levels in linking peripheral inflammation, central inflammation, and AD.

## Data Availability

Data used in this study are publicly available from the online ADNI Image and Data archive: https://ida.loni.usc.edu/login.jsp?project=ADNI. Data generated during additional processing and analysis are available from the corresponding author upon request.
